# Neuropsychological Correlates of Brain Perfusion SPECT in Patients with Macrophagic Myofasciitis

**DOI:** 10.1371/journal.pone.0128353

**Published:** 2015-06-01

**Authors:** Axel Van Der Gucht, Mehdi Aoun Sebaiti, Emmanuel Itti, Jessie Aouizerate, Eva Evangelista, Julia Chalaye, Romain K. Gherardi, Nilusha Ragunathan-Thangarajah, Anne-Catherine Bachoud-Levi, François-Jérôme Authier

**Affiliations:** 1 Department of Nuclear Medicine, H. Mondor Hospital, Assistance Publique-Hôpitaux de Paris/Paris-Est University, Créteil, F-94010, France; 2 Department of Neurology, H. Mondor Hospital, Assistance Publique-Hôpitaux de Paris/Paris-Est University, Créteil, F-94010, France; 3 Department of Pathology, H. Mondor Hospital, Assistance Publique-Hôpitaux de Paris/Paris-Est University, Créteil, F-94010, France; 4 Reference Center for Neuromuscular Disorders, H. Mondor Hospital, Assistance Publique-Hôpitaux de Paris, Créteil, F-94010, France; 5 INSERM U955-Team 10, Créteil, F-94010, France; 6 INSERM U955-Team 1, Créteil, F-94010, France; University of Würzburg, GERMANY

## Abstract

**Background:**

Patients with aluminum hydroxide adjuvant-induced macrophagic myofasciitis (MMF) complain of arthromyalgias, chronic fatigue and cognitive deficits. This study aimed to characterize brain perfusion in these patients.

**Methods:**

Brain perfusion SPECT was performed in 76 consecutive patients (aged 49±10 y) followed in the Garches-Necker-Mondor-Hendaye reference center for rare neuromuscular diseases. Images were acquired 30 min after intravenous injection of 925 MBq ^99m^Tc-ethylcysteinate dimer (ECD) at rest. All patients also underwent a comprehensive battery of neuropsychological tests, within 1.3±5.5 mo from SPECT. Statistical parametric maps (SPM12) were obtained for each test using linear regressions between each performance score and brain perfusion, with adjustment for age, sex, socio-cultural level and time delay between brain SPECT and neuropsychological testing.

**Results:**

SPM analysis revealed positive correlation between neuropsychological scores (mostly exploring executive functions) and brain perfusion in the posterior associative cortex, including cuneus/precuneus/occipital lingual areas, the periventricular white matter/corpus callosum, and the cerebellum, while negative correlation was found with amygdalo-hippocampal/entorhinal complexes. A positive correlation was also observed between brain perfusion and the posterior associative cortex when the time elapsed since last vaccine injection was investigated.

**Conclusions:**

Brain perfusion SPECT showed a pattern of cortical and subcortical changes in accordance with the MMF-associated cognitive disorder previously described. These results provide a neurobiological substrate for brain dysfunction in aluminum hydroxide adjuvant-induced MMF patients.

## Introduction

Macrophagic myofasciitis (MMF) is a longstanding inflammatory lesion found at deltoid muscle biopsy assessing persistence of aluminum hydroxide adjuvant particles within macrophages following intramuscular vaccine injections (#ORPHA592, http://www.orpha.net) [1,2]. Clinical manifestations associated with MMF typically include arthromyalgias and chronic fatigue, occurring several months or years after the last vaccine injection [3–5]. A majority of patients complain of cognitive dysfunction, combining impaired visual memory, dysexecutive syndrome and alteration of dichotic listening [6]. This MMF-associated cognitive dysfunction (MACD), is distinct from non-specific impairment observed in other arthromyalgic conditions and is not simply attributable to chronic pain, fatigue and depression. It resembles that observed upon chronic exposure to aluminum particles and in patients infected by hepatitis C virus or human immunodeficiency virus (HIV) [6]. At follow-up, it appears stable over time, both in structure and severity [7].

This cognitive dysfunction suggests underlying cortico-subcortical brain lesions, possibly of inflammatory or toxic origin. Experimental data evidenced that after parenteral injections of aluminum hydroxide, aluminum particles can translocate into the brain tissue where they remain trapped [8–11]. Inorganic particulate materials can be transported by monocyte-lineage cells to distant tissues, and, similarly to HIV, may use MCP1-dependent monocyte transmigration across the blood-brain barrier to enter the brain [11,12]. Interestingly, patients with MMF display selective increase of circulating MCP1 levels, which could make them prone to progressively accumulate aluminum in brain and develop low-noise neurotoxicity [13]. However, to date, there is no pathological or radiological evidence for brain damages specifically associated with MMF [14]. In particular, except in the subset of patients who have co-occurring multiple sclerosis, standard MR brain imaging failed to explain cognitive dysfunctions in MMF, and, to our knowledge, no post-mortem brain examination of MMF patient has been reported so far.

Brain perfusion SPECT is routinely used to evaluate cognitive dysfunctions. Purpose of the present exploratory study was to investigate whether a substrate of brain perfusion may be identified, in correlation with neurospychological alteration in a large series of MMF patients with varying degrees of MACD.

## Materials and Methods

### Patients

The study population included 76 consecutive patients with histopathological features of MMF at muscle biopsy and a cognitive complain, followed in the Garches-Necker-Mondor-Hendaye reference center for rare neuromuscular diseases. Mean age was 49 ± 10 y, sex ratio 21 men/55 women, and mean socio-cultural level (years of education after highschool) 4.9 ± 1.3 y. All but 4 patients were right handed. Patients presented with MACD of varying severity and underwent both brain perfusion SPECT and neuropsychological testing within 1.3 ± 5.5 mo from each other, as standard care and in accordance with current national regulations (verbal informed consent was obtained and reported in patient medical records). Collection and analysis of data was retrospective and performed after de-identification. This non-interventional study was approved by our IRB in its present form on December 18, 2013 (Comité de Protection des Personnes Ile-de-France VI), and written consent was waived.

### Brain perfusion scans

Patients underwent brain perfusion SPECT 30 min after intravenous injection of 740 MBq ^99m^Tc-ethylcysteinate dimer (ECD), at rest, in a dimly lit room. Images were acquired on a dual-headed Axis γ-camera (Philips, DaBest, The Netherlands) equipped with high-resolution collimators. They consisted of 120 projections of 14 s each and were reconstructed into transaxial image volumes using an ordered subset expectation maximization algorithm with 4 iterations, followed by Butterworth filtering (order 4, cutoff 0.35 cycles/pixel), and Chang attenuation correction.

### Neuropsychological Testing

Patients underwent a comprehensive battery of neuropsychological tests exploring specific MACD domains (Table 1). Assessment of executive functions included tests exploring, working memory (backward digit span, letter-number sequencing part of the Wechsler adult intelligence scale [WAIS-III], the Brown-Peterson paradigm, and Zazzo cancellation tests), flexibility (Trail Making Test B), inhibition (Stroop test) and planning (Rey-Osterrieth complex figure copy and image arrangement of the WAIS-III). Attention was explored by the Zazzo cancellation tests. Short-term visual memory was assessed the Benton Visual Retention Test and long-term visual memory by the Rey-Osterrieth delayed recall. Immediate verbal memory was assessed using the forward digit span, and episodic verbal memory Grober & Buschke long-term free (recovery capacity) and cued (storage capacity) recall. Finally, interhemispheric connection was tested by dichotic listening of words and sentences tasks, successively.

**Table 1:** Patient performance to neuropsychological tests.

Characteristics		Patient number	Raw scores (mean ± SD)	Normalized scores * (mean ± SD)
***Executive functions***	**WAIS-III matrix**	74	8.97 ± 2.47	-0.35 ± 0.83
	**WAIS-III pictures**	64	8.94 ± 2.81	-0.35 ± 0.93
	**WAIS-III letter-number sequencing**	62	8.24 ± 2.42	-0.57 ± 0.81
	**Brown-Peterson 0**	35	95.59 ± 11.12	-2.28 ± 4.25
	**Brown-Peterson 5**	35	83.31 ± 21.19	-0.94 ± 1.83
	**Brown-Peterson 10**	35	72.06 ± 25.31	-0.94 ± 1.91
	**Brown-Peterson 20**	35	67.83 ± 28.36	-0.85 ± 1.79
	**Stroop color and word**	51	42.80 ± 9.61	-0.68 ± 0.98
	**Stroop interferences**	50	47.22 ± 6.80	-0.21 ± 0.71
	**RO copy**	76	33.78 ± 2.23	-0.77 ± 1.55
	**RO delayed recall**	75	19.51 ± 5.71	-0.46 ± 1.02
***Verbal memory***	**Forward digit span**	76	5.93 ± 1.28	-0.09 ± 1.15
	**Backward digit span**	76	4.30 ± 1.22	-0.31 ± 1.33
***Memory functions***	**Grober & Buschke-LTFR**	50	11.38 ± 3.53	-0.68 ± 1.90
	**Grober & Buschke-LTCR**	49	15.20 ± 1.46	-1.52 ± 4.31
***Visual memory***	**Benton visual retention test**	62	18.54 ± 3.22	-0.75 ± 1.53
***Flexibility***	**Trail making test B**	73	104.84 ± 62.85	2.46 ± 3.99
***Callosal disconnection***	**DL left ear words**	73	53.16 ± 11.24	2.75 ± 10.99
	**DL right ear words**	73	53.07 ± 8.63	0.44 ± 8.37
	**DL left ear sentences**	72	15.65 ± 4.34	1.63 ± 4.38
	**DL right ear sentences**	72	17.38 ± 3.04	2.19 ± 4.21
***Attention***	**Zazzo 1 sign**	58	54.01 ± 14.00	-0.71 ± 0.98
	**Zazzo 2 signs**	58	52.12 ± 13.78	-0.82 ± 0.96
	**Zazzo 3 signs**	58	38.71 ± 12.55	-1.16 ± 1.11
***Depression***	**Beck depression inventory II**	36	6.17 ± 9.26	-

* all values are expressed as Z-scores (-1.65 SD of the normal average), except for dichotic listening tasks (expressed as number of words or sentences compared to controls)

RO, Rey-Osterrieth; LTFR, Long-term free recall; LTCR, Long-term cued recall; DL, Dichotic listening;

Raw performance scores were converted into Z-scores by comparison with a control population in order to express pathological threshold at -1.65 standard deviations of the normal average, as previously described [6]. Therefore, a Z-score ≥ 0 indicated good performance (equal or better than normal), while a Z-score < 0 indicated poor performance. For dichotic listening tasks, the normalized scores expressed the number of words or sentences heard in comparison with the control population. The list of test references is provided in S1 list. In order to assess the influence of depression on cognitive performance, a Pearson correlation was performed between each Z-score and the Beck Depression Inventory II.

### Analysis of Statistical Parametric Maps (SPM)

All SPECT image volumes were spatially normalized onto the Montreal Neurological Institute (MNI) template (McGill University, Montreal, Canada) using a 12-parameter affine transformation, followed by non-linear transformations. Dimensions of the resulting voxels were 2 x 2 x 2 mm3. Images were smoothed using a Gaussian filter (FWHM 10 mm) to blur individual variations in anatomy and to increase the signal-to-noise ratio. Spatial preprocessing and statistical analysis were performed using the SPM12 software implemented in Matlab version R2014a (Mathworks Inc., Sherborn, MA). Global normalization was performed using proportional scaling to control for individual variation. Analyses were restricted to white matter when callosal disconnection was investigated and to gray matter for all other parameters using mask from WFU PickAtlas v.3 software (ANSIR Laboratory, Wake Forest University School of Medicine, Winston-Salem, NC). Statistical parametric maps were obtained, for each neuropsychological test, by linear regressions comparing brain perfusion in patients with their respective normalized scores, with adjustment for age, sex, socio-cultural level and time delay between brain SPECT and neuropsychological testing, these nuisance variables being known to have influence on brain perfusion. The effect of the time elapsed since last vaccine injection, as well as the number of vaccine injections, on regional brain perfusion was also investigated, using the same design. Results of linear regressions were collected at a *P*-value < 0.005 at the voxel level, for clusters k ≥ 100 voxels (corrected for cluster volume) in order to highlight correlation trends between regional brain perfusion and neuropsychological performance. Spearman correlation was used to study the relationship between extracted clusters of perfusion and neuropsychological scores. All significant results were listed with the individual k-value, which represents the number of significant voxels in the particular cluster (cluster size), brain regions, the side of the hemisphere, the Brodmann area (BA) involved and the peak T-value.

## Results

### Clinical features

Chronic myalgias and fatigue were found in 72 (94.7%) and 62 (81.6%) patients, respectively. The mean number of aluminum hydroxide-containing vaccines administered to patients within the past 10 years was 4.5 (range, 3–10 injections). The mean delay elapsed from the last aluminum-containing vaccine injection to muscle biopsy was 6.4 y (range, 1–14 y). Patient performances on neuropsychological tests are displayed in Table 1. All tests but one (Brown-Peterson, 0-second condition) were found independent from the level of depression assessed by the Beck Depression Inventory II.

### MMF-associated brain perfusion changes

**Fig 1:**
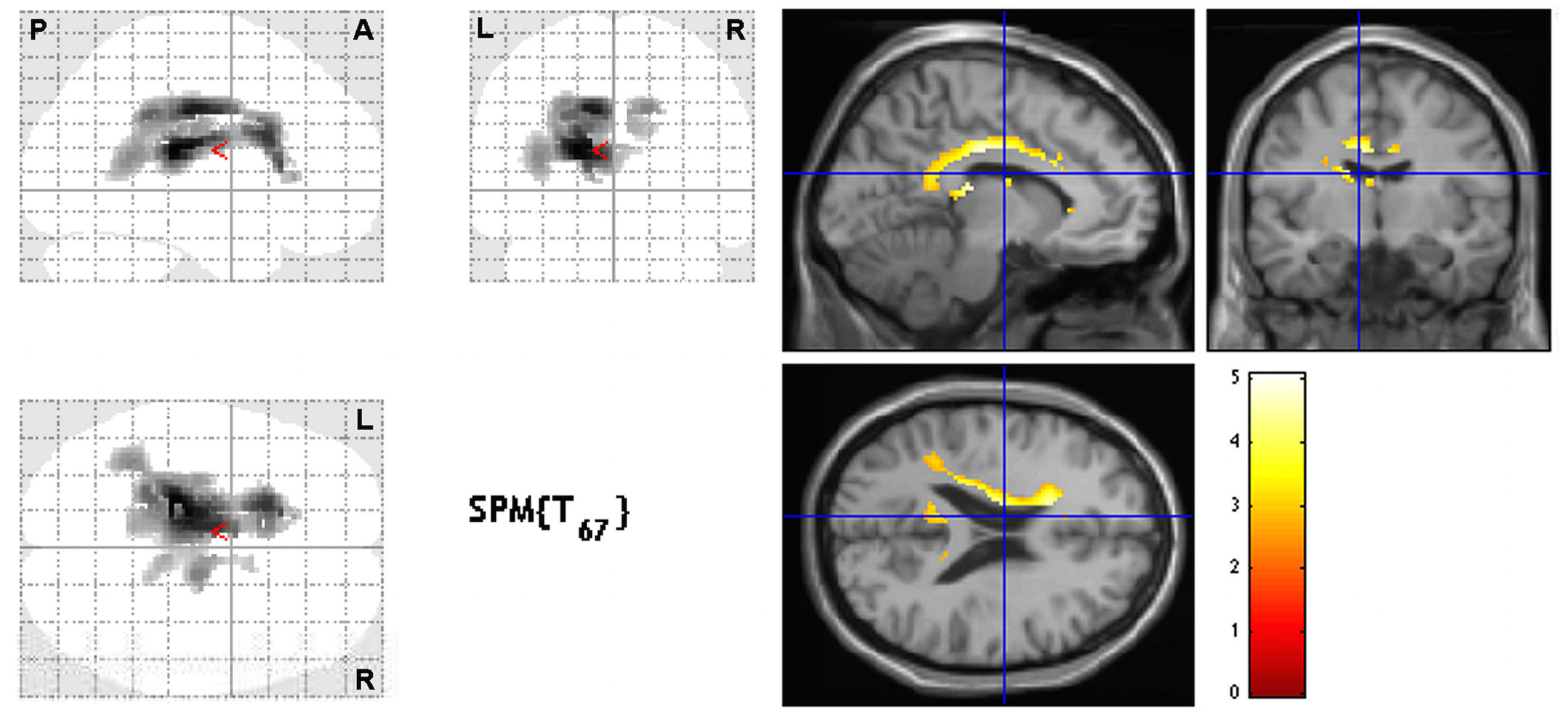
SPM map. Positive correlation between brain perfusion and dichotic listening (left ear words) normalized scores with adjustment for age, sex, socio-cultural level and time delay between brain SPECT and neuropsychological testing. Linear regression analysis shows diffuse impairment of periventricular areas/corpus callosum. Significant clusters are displayed with T-score values on 2-dimensional projections (glass-brain—left panel) and slices of MRI (right panel) templates in axial, coronal and sagittal orientations. *P*-value < 0.005 at the voxel level for clusters ≥ 100 contiguous voxels (corrected for cluster volume). L, left; R, right; A, anterior; P, posterior.

Brain areas which showed positive or negative correlation trends with the performance on tests are displayed in Table 2. The most significant finding was that dichotic listening impairment to the left ear (words and sentences) was positively correlated with perfusion in the periventricular areas and corpus callosum (Fig 1, white matter mask). SPM12 map of left-ear words correlation revealed a single large cluster of 2180 voxels with the highest peak T-value of all linear regressions (5.09), involving the white matter of both left and right hemispheres, and, to a lesser extent, SPM12 map of left-ear sentences revealed 2 clusters covering the same areas (sum, 1877 voxels).

**Table 2:** Linear regression analyses of cognitive scores and time since last vaccine injection, with adjustment for age, sex, socio-cultural level and delay between brain SPECT and neuropsychological testing.

Characteristics		K	Brain areas	Side	Labels	P-value	Peak T-value
***Executive functions***	**WAIS-III matrix**	-	-	-	-	-	-
	**WAIS-III pictures**	-	-	-	-	-	-
	**WAIS-III letter-number sequencing**	181	Temporal lobe	R	BA21, BA22, BA42, BA20	0.002	4.76
		197	Cerebellum	R	Cerebellum Anterior Lobe	<0.001	4.74
		143	Parietal lobe	L	BA7	0.001	4.14
		244	Parietal lobe, frontal lobe	R	BA2, BA3, BA40, BA1, BA4	0.001	4.09
		180	Occipital lobe	R	BA18, BA19, BA17	0.001	3.90
		122	Frontal lobe	R	BA9, BA8, BA46	<0.001	3.52
		274	Sub-lobar	L	Thalamic nucleus	0.001	3.50
		159	Cerebellum	R	Cerebellum posterior lobe	0.002	3.35
	**Brown-Peterson 0**	331	Cerebellum	L	Cerebellum posterior lobe	0.002	4.61
	**Brown-Peterson 5**	-	-	-	-	-	-
	**Brown-Peterson 10**	395*	Limbic lobe, sub-lobar, temporal lobe	L	Amygdalo-hippocampal, BA28, BA34, striatum, BA38	0.001	4.32
	**Brown-Peterson 20**	-	-	-	-	-	-
	**Stroop color and word**	247	Occipital lobe	R	BA18, BA19	0.001	4.66
		203	Occipital lobe	L	BA18, BA19, BA17	<0.001	4.52
		174	Parietal lobe	R	BA40, BA7	<0.001	4.12
		164	Parietal lobe, frontal lobe	R	BA40, BA3, BA2, BA4	0.001	3.60
		202	Parietal lobe	L	BA7, BA40	0.001	3.56
		423*	Limbic lobe	L	Amygdalo-hippocampal, BA28, BA34, BA35, BA36, BA20	0.003	4.18
	**Stroop interferences**	-	-	-	-	-	-
	**RO copy**	-	-	-	-	-	-
	**RO delayed recall**	168	Occipital lobe	L	BA18, BA19	0.001	4.31
***Verbal memory***	**Forward digit span**	223*	Occipital lobe, parietal lobe	L	BA18, BA19, BA31, BA7	0.001	3.92
		134*	Occipital lobe, parietal lobe, limbic lobe	R	BA18, BA19, BA31, BA17	0.001	3.24
	**Backward digit span**	-	-	-	-	-	-
***Memory functions***	**Grober & Buschke-LTFR**	-	-	-	-	-	-
	**Grober & Buschke-LTCR**	-	-	-	-	-	-
***Visual memory***	**Benton visual retention test**	-	-	-	-	-	-
***Flexibility***	**Trail making test B**	-	-	-	-	-	-
***Callosal disconnection***	**DL left ear words**	2180	Sub-lobar, limbic lobe, frontal lobe	L, R	Periventricular areas, corpus callosum	0.003	5.09
	**DL right ear words**	270*	Cerebellum	L, R	Cerebellum posterior lobe, cerebellum anterior lobe	0.001	3.50
	**DL left ear sentences**	1354	Sub-lobar, limbic lobe, frontal lobe	R	Periventricular areas, corpus callosum	0.003	4.45
		523	Sub-lobar, limbic lobe, frontal lobe	L	Periventricular areas, corpus callosum	0.001	4.18
		519*	Cerebellum	L	Cerebellum posterior lobe	0.001	4.27
	**DL right ear sentences**	175*	Limbic lobe, temporal lobe	R	Amygdalo-hippocampal, BA28, BA34, BA38	0.002	3.96
***Attention***	**Zazzo 1 sign**	-	-	-	-	-	-
	**Zazzo 2 signs**	-	-	-	-	-	-
	**Zazzo 3 signs**	-	-	-	-	-	-
***Depression***	**Beck Depression Inventory II**	-	-	-	-	-	-
***Time since last vaccine injection***		112	Occipital lobe, temporal lobe	R	BA19, BA39	0.001	3.67

* Indicates negative correlation (the lower the score, the higher the perfusion)

*P*-value < 0.005 at the voxel level for clusters ≥ 100 contiguous voxels (corrected for cluster volume).

BA, Brodmann areas; R, right; L, left; RO, Rey-Osterrieth; LTFR, Long-term free recall; LTCR, Long-term cued recall; DL, Dichotic listening

**Fig 2:**
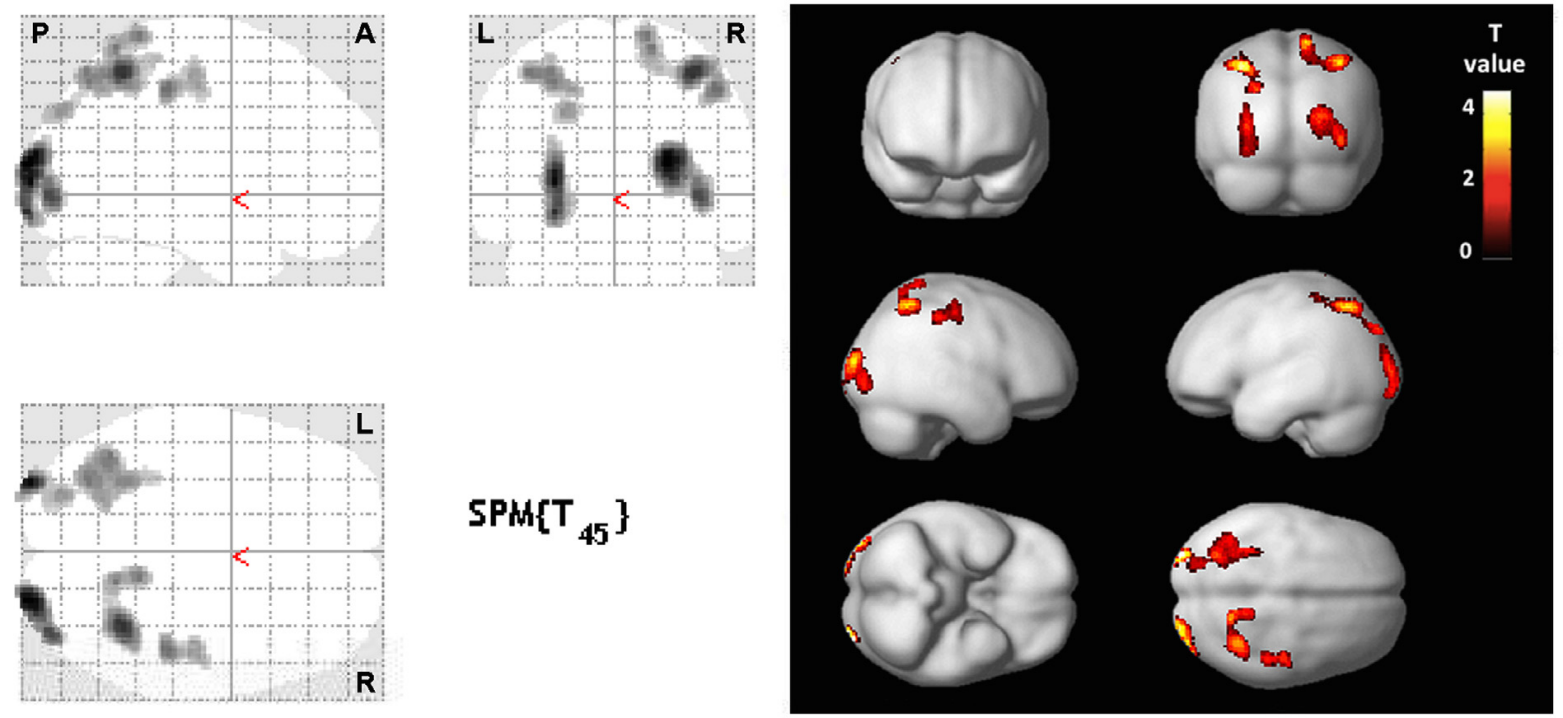
SPM map. Positive correlation between brain perfusion and Stroop color and word Z-scores with adjustment for age, sex, socio-cultural level and time delay between brain SPECT and neuropsychological testing. Linear regression analysis shows impairment of posterior associative areas. Significant clusters are displayed with T-score values on 2-dimensional axial, coronal and sagittal orientations (glass-brain—left panel) and projected onto a brain rendered 3D MIP (right panel). P-value < 0.005 at the voxel level for clusters ≥ 100 contiguous voxels (corrected for cluster volume). L, left; R, right; A, anterior; P, posterior.

With regards to gray matter perfusion, most positive correlations were found between tests exploring executive functions and perfusion in the posterior associative regions (Fig 2), including cuneus/precuneus (BA7/BA17 and WAIS-III/Stroop tests) and occipital lingual areas (BA18/BA19 and WAIS-III/Stroop/Rey-Osterrieth recall tests). Few correlations were found between these tests and perfusion in the frontal or temporal lobes. Conversely, tests exploring executive functions were inversely correlated with perfusion in the limbic system, including left amygdalo-hippocampal/entorhinal complex (BA28/BA34 and Brown-Peterson 10/Stroop). Negative correlation was also found between tests exploring verbal memory (forward digit span) and the posterior associative areas (occipital, BA18/BA19; posterior cingulate, BA31).

Perfusion in the cerebellum was positively correlated with tests exploring executive functions (WAIS-III/Brown-Peterson). To note, perfusion in the right hippocampus was inversely correlated with dichotic listening to the right ear. Finally, a positive correlation was noted between brain perfusion in the right posterior associative cortex (BA19, BA39) and the time elapsed since last vaccine injection. No significant correlation was observed between brain perfusion and the number of vaccine injections.

## Discussion

This exploratory study showed diffuse cortical and subcortical changes associated with cognitive impairment in patients with MMF. The pattern of significant clusters revealed by SPM12 analysis in this large population of patients with varying disease severities provides a neurobiological substrate for brain dysfunction in aluminum hydroxide-induced MMF patients. From a neuropsychological point of view, we observed a MACD profile similar to that of previously published data, combining dysexecutive syndrome, impaired memory, and alteration of dichotic listening [6,7]. Performance on tests was independent from the depression level (except Brown Peterson 0) and therefore, the bad scores cannot be attributed to a depressive state. It has to be noted that all neuropsychological test were not available in all patients as some patients could not complete the full battery due to marked fatigability.

Linear regression analyses emphasized diffuse involvement of periventricular white matter and corpus callosum, proportional to the impairment to left ear dichotic listening tasks. White matter perfusion is generally difficult to assess on an individual level because most of the tracer takes up on the gray matter with a gray-to-white ratio > 2 [15]. However, involvement of periventricular white matter was highly significant with SPM12 analysis in this large population. Although most patients performed well on dichotic listening (mean score for the whole population = +2.75 words compared with normal), a subset of 12 patients (16%) with callosal disconnection performed very poorly (mean score, -16 words compared with normal), which explains why this positive correlation was our most significant findings. These structures warrant the connection between right and left brain hemispheres through the corpus callosum [16]. Diffusion tensor imaging has confirmed that stronger anatomical connection between right and left temporal lobe auditory processing areas supports a better information transfer [17]. They are also involved in frontal subcortical loops responsible for dysexecutive syndrome [18], which is one of the predominant features of MACD. Our study demonstrates the specific involvement of these subcortical pathways, but also in their cortical afferences (posterior associative areas).

With regards to gray matter involvement, most of the positive correlations were found between tests exploring executive functions and posterior associative areas, including BA18/BA19 (secondary and tertiary visual cortex) and BA5/BA7/BA17 (cuneus/precuneus). These regions are frequently involved in neurodegenerative diseases, such as Alzheimer or Parkinson's dementias [19,20]. However, the posterior cingulate gyrus would also be impaired, while it was spared in patients with MMF, as shown by the inverse correlation found between BA31 and forward digit span. As a fact, verbal memory performances are known to be preserved in MACD [6] and cognitive dysfunction is stable with time [7], which differs from neurodegenerative disorders. Finally, a direct relationship was found between the time from last vaccine injection and perfusion in the posterior associative areas, which supports the idea that MACD does not worsen with time.

Cerebellum is known to be involved in motor functions. It has also an important role in cognitive processing, particularly in executive functions [21]. This activity is due to the numerous connections between the cerebellum and cortical areas through the cortico-ponto-cerebellar pathways [22]. Afferent fibers mostly come from associative parietal and occipital areas, which are significantly impaired in our study. Limbic system, including amygdalo-hippocampal/entorhinal complexes and cingulate gyrus, also play an essential role in long-term memory storage, and are involved in the Papez circuit [23]. These structures are the first to be impaired in mild cognitive impairment conversion into Alzheimer's disease [24]. Interestingly, we found a significant inverse correlation between left amygdalo-hippocampal perfusion and executive functions (Brown-Peterson 10/Stroop), and between right amygdalo-hippocampal perfusion and dichotic listening (right ear sentences, Table 2). It can be hypothesized that some patients may compensate hippocampal dysfunction by contralateral activation, while those who have callosal disconnection cannot. As a fact, patients with callosal disconnection had significant lower Z-scores on delayed free recall (-2.56 ± 2.46 vs. -0.27 ± 1.59, *P* = 0.002) and delayed cued recall (-6.42 ± 8.32 vs. -0.43 ± 1.92, *P* = 0.0002) tasks.

It is important to rely on pretest diagnostic indices before performing an invasive procedure such as deltoid muscle biopsy. Among arthromyalgic patients with both history of immunization by aluminum hydroxide-containing vaccines and deltoid muscle biopsy, a minority have MMF; they differ from non-MMF patients both by much more neurological dysfunction and much less fibromyalgic tender points [25]. Consistently, the present study shows that the cerebral perfusion pattern in patients with MMF differs from that reported in fibromyalgia [26]. Both conditions share hypoperfusions of temporal lobes, cingulate gyrus and cerebellum, but, in addition, patients with MMF typically show involvement of periventricular and posterior associative areas, which are either preserved or show hyperperfusion, respectively, in fibromyalgia. Therefore, brain perfusion imaging may help distinguishing patients with MMF from those with common fibromyalgia.

## Conclusion

Brain perfusion imaging showed a pattern of diffuse cortical and subcortical changes, in accordance with the MMF-associated cognitive disorder previously described. These results provide a neurobiological substrate for brain dysfunction in aluminum hydroxide-induced MMF patients and may warrant further studies using metabolic imaging and diffusion MRI to assess possible neuronal damage.

## Supporting Information

S1 List References list of neuropsychological tests. (DOC)
