# Supplementary material for: Neuropsychological Correlates of Brain Perfusion SPECT in Patients with Macrophagic Myofasciitis
Source: PLoS One. 2015 Jun 1;10(6):e0128353. doi: 10.1371/journal.pone.0128353 (PMC4451975; doi:10.1371/journal.pone.0128353)
Supplement: S1 List — (DOC) [file pone.0128353.s001.doc]

Benton AL. Revised Visual Retention Test, 4th Edition. Psychological Corporation, New York, 1974.

Osterrieth PA. Le test de copie d’une figure complexe: contribution à l’étude de la perception et de la mémoire. Arch Psychol. 1944;30:286–356.

Stroop JR. Studies of interference in serial verbal reactions. J Exp Psychol. 1935;18:643–661.

Golden C. Stroop color and word test. Illinois: Stoelting Company, 1978.

Wechsler D. Echelle d'intelligence de Wechsler pour adultes WAIS-III. Les Editions du Centre de Psychologie Appliquée, Paris, 2000.

Wechsler D. Echelle de mémoire de Wechsler MEM III. Les éditions du Centre de Psychologie appliquée, Paris, 2001.

Van Der Linden M, Coyette F, Poitrenaud J, et al. L'épreuve de rappel libre / rappel indicé à 16 items (RL/RI16). In: Van Der Linden M, Adam S, Agniel A, et al. (Eds.), L'évaluation des troubles de la mémoire épisodique (avec leur étalonnage) 2004, pp. 25-48. Marseille: Solal.

Grober E, Buschke H. Genuine memory deficits in dementia. Develop Neuropsychol. 1987;3:13-36.

Army Individual Test Battery. Manual of Directions and Scoring. War Department, Adjutant General's Office, Washington DC, 1944.

Tombaugh TN. Trail Making Test A and B: Normative data stratified by age and education. Arch Clin Neuropsychol. 2004;19:203-214.

Brown J. Some tests of the decay theory of immediate memory. Q J Exp Psychol. 1958;10:12-21.

Zazzo R. Manuel pour l’examen psychologique de l’enfant (3e éd.). Delachaux & Niestlé, Neuchâtel, 1972.

Kimura D. Some effects of temporal-lobe damage on auditory perception. Can J Exp Psychol. 1961;15:156-165.

Peterson LR, Peterson MJ. Short-term retention of individual verbal items. J Exp Psychol. 1959;58:193-198.
